# Strain-Induced Ferromagnetism in Monolayer T″-Phase VTe_2_: Unveiling Magnetic States and Anisotropy for Spintronics Advancement

**DOI:** 10.3390/nano14080704

**Published:** 2024-04-18

**Authors:** Xiaoting Tang, Jun Zhou, Nancy Lai Mun Wong, Jianwei Chai, Yi Liu, Shijie Wang, Xiaohe Song

**Affiliations:** 1 Department of Physics, College of Science, Shanghai University, 99 Shangda Road, Shanghai 200444, China; tangxiaoting@shu.edu.cn; 2 Department of Physics, National University of Singapore, Singapore 117542, Singapore; 3 Institute of Materials Research and Engineering (IMRE), Agency for Science, Technology and Research (A*STAR), 2 Fusionopolis Way, Innovis #08-03, Singapore 138634, Singapore; zhou_jun@imre.a-star.edu.sg (J.Z.); wongn@imre.a-star.edu.sg (N.L.M.W.); jw-chai@imre.a-star.edu.sg (J.C.); 4 Materials Genome Institute (MGI), Shanghai University, 333 Nanchen Road, Shanghai 200444, China; 5 EACOMP, Shenzhen 518055, China

**Keywords:** 2D magnet, charge density wave, strain, magnetic anisotropy, VTe_2_, Hubbard U

## Abstract

Two-dimensional (2D) ferromagnets have attracted significant interest for their potential in spintronic device miniaturization, especially since the discovery of ferromagnetic ordering in monolayer materials such as CrI_3_ and Fe_3_GeTe_2_ in 2017. This study presents a detailed investigation into the effects of the Hubbard U parameter, biaxial strain, and structural distortions on the magnetic characteristics of T″-phase VTe_2_. We demonstrate that setting the Hubbard U to 0 eV provides an accurate representation of the observed structural, magnetic, and electronic features for both bulk and monolayer T″-phase VTe_2_. The application of strain reveals two distinct ferromagnetic states in the monolayer T″-phase VTe_2_, each characterized by minor structural differences, but notably different magnetic moments. The T″-1 state, with reduced magnetic moments, emerges under compressive strain, while the T″-2 state, featuring increased magnetic moments, develops under tensile strain. Our analysis also compares the magnetic anisotropy between the T and T″ phases of VTe_2_, highlighting that the periodic lattice distortion in the T″-phase induces an in-plane anisotropy, which makes it a material with an easy-axis of magnetization. Monte Carlo simulations corroborate our findings, indicating a high Curie temperature of approximately 191 K for the T″-phase VTe_2_. Our research not only sheds light on the critical aspects of the VTe_2_ system but also suggests new pathways for enhancing low-dimensional magnetism, contributing to the advancement of spintronics and straintronics.

## 1. Introduction

The experimental confirmation of two-dimensional (2D) long-range ferromagnetic (FM) ordering in materials like CrI_3_ and CrGeTe_3_ has sparked considerable research interest in the realm of low-dimensional magnetism, serving both fundamental physics exploration and practical applications [1,2,3,4,5,6]. Subsequently, numerous other 2D ferromagnets have been reported using different methodologies, including high-throughput screening and machine learning [7,8,9,10,11,12,13]. The unexpected revelation of such long-range 2D magnetic ordering caused a stir in the materials community, as the Mermin–Wagner theorem had previously deemed any low-dimensional long-range ordering impossible at finite temperatures [14]. Further investigations have elucidated that magnetic anisotropy can indeed stabilize long-range magnetism in 2D materials [15]. Alongside the challenging quest for the rare intrinsic magnetic easy axis in 2D magnets, material engineering techniques, such as inducing magnetic anisotropy through uniaxial strain in initially easy-plane magnetic systems, have been successfully employed [16,17,18].

Transition metal dichalcogenides (TMDCs) represent a rich repository of 2D ferromagnets. The magnetic moments observed in TMDCs stem from the partial filling of *d* orbitals in transition metal ions, facilitating the possibility of long-range magnetic order under specific conditions. Moreover, the presence of heavy elements such as Se and Te impart considerable spin–orbit coupling (SOC) to TMDCs. Notably, tellurides are particularly appealing due to the lower electronegativity and enhanced SOC effects of Te atoms [19]. For instance, group V metal tellurides (VTe_2_, NbTe_2_, TaTe_2_) showcase diverse structural variations and hold promise as a platform for investigating various fundamental physics phenomena, including superconductivity, electro-catalytic activity, excitons, and the quantum spin Hall effect [20]. Experimental studies by Mitsuishi et al. have confirmed the existence of topological surface states in both T and T″ bulk VTe_2_ [21,22]. Furthermore, the prospect of ferromagnetic TMDCs opens up avenues for further exploration in fundamental physics and the development of novel magneto-electronic/optoelectronic devices [23].

**Scheme 1:**
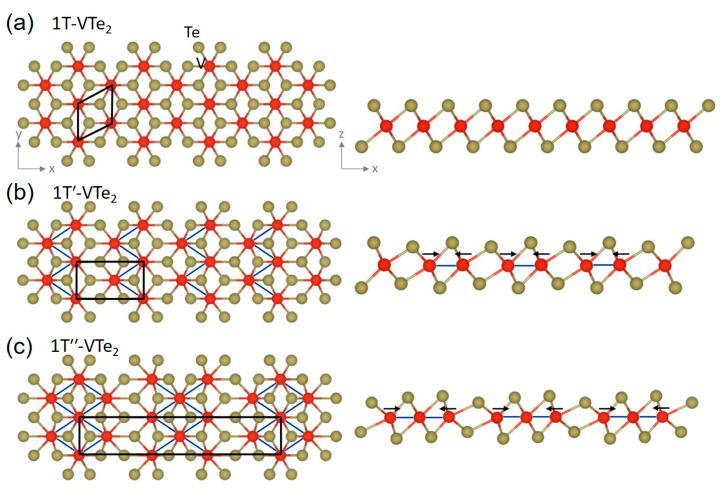
The structures of (**a**) T-phase, (**b**) T′-phase, and (**c**) T″-phase VTe_2_. The red and brown balls represent the V and Te atoms, respectively. The black rhombus and rectangles are the unit cells for the three phases. Each subplot includes a top view on the left and a side view on the right. In the T′-phase VTe_2_, single zigzag chains (indicated by blue lines in (**b**)) are formed by the head-to-head movement of the V pairs, while the T″ phase is a 3 × 1 reconstruction with the two V atoms on the two sides moving head-to-head towards the middle V atom, forming double zigzag chains (indicated by blues in (**c**)).

An intriguing physical phenomenon within TMDCs is the charge density wave (CDW), which draws considerable attention due to its wide existence, rich patterns, and capability to be tunable by external influences [24,25,26,27,28,29]. CDWs are periodic modulations of charge density by lattice distortions in real space, alongside the opening of a band gap, below a critical temperature [30]. The CDW patterns in TMDCs have been proposed to depend critically on the *d*-electron count of the transition metal ions. For example, the most common CDW configurations include the $\sqrt{13}\times \sqrt{13}$, 3×1, 2×1, and 2×2 rearrangements of metal ions for TMDCs with *d*^1^, *d*^1+*x*^ (0 < *x* < 1), *d*^2^ and *d*^3^ TM ions, respectively [24]. The more related CDW phases for VTe_2_ are the T′ and T″ phases. As shown in Scheme 1, the T′ phase is characterized by a 2×1 reconstruction of the unit cell in the T phase, forming single zigzag chains, and the T″ phase is a 3×1 reconstruction with the two V atoms at the two sides moving head-to-head to the middle V atom, forming ribbon or double zigzag chains. The presence of CDWs can significantly alter a material’s properties, such as its mutual suppression with magnetism [31,32,33,34,35,36]. However, recent findings also suggest that CDW transitions can promote the formation of magnetism [37]. Thus, the interplay between CDWs and magnetism remains a subject of ongoing study, with the precise nature of their interaction still being elusive.

Despite this, VTe_2_ stands out for its diverse phase variants and magnetic properties. In its bulk form, VTe_2_ assumes the high-symmetric T phase at high temperatures, but undergoes a 3 × 1 charge density wave (CDW) reconstruction, which is named T″ phase in the later sections, at a critical temperature of approximately $T_{CDW}\approx \;480$ K. Within the temperature range of 30–480 K, the T″-like CDW phases of bulk VTe_2_ are reported to exhibit two types of antiferromagnetic (AFM) behavior [38]. As bulk VTe_2_ is gradually thinned down to thin film or even monolayer, some fascinating phase evolutions appear. For instance, starting from the T″ in bulk, the phase transforms into the T phase in multilayer thin films, then to the T′ in bilayers, and ultimately to the T phase in monolayers [23,39,40]. Both the T′ and T″ phases are close variants of the T phase. Presently, no long-range magnetic order has been observed in monolayer T-phase VTe_2_ [41,42], although ferromagnetic behavior has been detected in multilayer T-phase VTe_2_ [23]. Considering the complex coupling among the charge, lattice, and spin order of VTe_2_, a close examination of its properties is imperative.

In this work, we conducted a thorough investigation into the multifaceted structural, electronic, and magnetic characteristics of VTe_2_, considering the impacts of both simulation parameters and structural modifications. Notably, our findings reveal that the periodic distortion present in VTe_2_ (T″ phase) can induce magnetic anisotropy and effectively stabilize two-dimensional magnetism, even up to a high Curie temperature. These results not only deepen our understanding of VTe_2_ but also present a promising pathway for enhancing low-dimensional magnetism. By elucidating the intricate interplay between structural distortions and magnetic properties, our study opens up new avenues for advancing the field of low-dimensional magnetism, potentially catalyzing the development of novel magnetic materials for future technological applications.

## 2. Materials and Methods

The first-principles calculations were performed based on the projector augmented-wave method as implemented in the Vienna ab initio simulation package (VASP 5.4.4) [43,44,45]. The exchange–correlation interaction was treated with the Perdew–Burke–Ernzerhof form of the generalized gradient approximation (GGA) [46]. The plane-wave cutoff energy was set to 500 eV. The lattice parameters and atomic positions were fully relaxed until the energy and force on each atom converged to less than 1 × 10^−5^ eV and 1 × 10^−3^ eV/Å, respectively. For magnetic anisotropy energy, the spin–orbital coupling was included with a higher cutoff criterion of 1 × 10^−8^ eV for the energy convergence. The K-mesh density was determined following the rule that *a_i_* × *k_i_* ≈ 50, where *a_i_* (unit Å) is the lattice parameter of lattice vector *i* (*i* = 1, 2, 3), and *k_i_* is the number of K-points corresponding to lattice vector *i*.

The VAMPIRE 5.0 package was used for metropolis Monte Carlo (MC) simulation [47,48], which was based on the following Heisenberg spin Hamiltonian:

(1) $$\tilde{H}=\sum_{ij}J_{ij}S_{i}\cdot S_{j}-A\sum_{i}S_{iz\;}^{2}$$

where $S_{i}$ and $S_{j}$ are the classical Heisenberg spins with unit magnitude at the site *i* and *j*. *J* is the nearest neighboring spin–spin coupling, and *A* is the magnetic anisotropic energy. The simulated spin systems for VTe_2_ consisted of 9600 spins, which were large enough to derive the converged magnetization–temperature curve. At each temperature, the orientations of the spins were initially randomized, and all the spins were first thermalized for 10,000 equilibrium steps and then by 50,000 averaging steps to derive the thermal equilibrium magnetization.

## 3. Results

### 3.1. The Determination of the Hubbard U Value for VTe_2_

The determination of the Hubbard U value holds paramount importance in ensuring the accuracy of Density Functional Theory (DFT) simulations, especially within vanadium-included compounds characterized by moderate correlation, which poses challenges to the determination process. Thus, it is imperative to exercise caution when evaluating its impacts on both crystalline and electronic structures. This section aims to elucidate our selection of the Hubbard U value, considering its implications on both crystalline and electronic structures.

**Figure 1:**
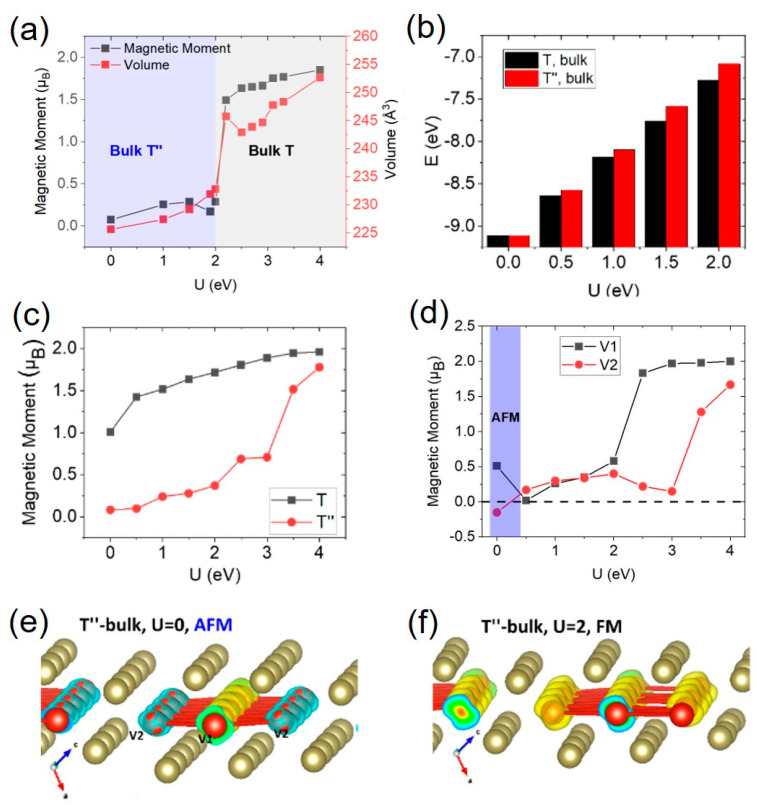
The evolution of preferred phase, relative phase stability, and magnetic order in bulk VTe_2_ as a function of U. (**a**) Evolution of the crystalline phase, magnetic moment per V atom, and volume per three VTe_2_ units as a function of U values, with bulk T″-phase VTe_2_ as the starting point for the fully structural relaxation in DFT calculations. (**b**) The relative stability of bulk T″-phase VTe_2_ versus bulk T-phase VTe_2_ at different U values. (**c**) Comparison of the calculated average magnetic moments for bulk T−- and bulk T″-phase VTe_2_ with fixed atomic positions during DFT calculations. (**d**) The magnetic moment of V atoms (V1 and V2 denote two types of V atoms shown in (**e**)) in the T″-phase VTe_2_ bulk at different U. The AFM and FM within the image refer to the magnetic configurations illustrated in (**e**,**f**). (**e**,**f**) illustrate the magnetic ordering in each layer of bulk T″-phase VTe_2_ at U = 0 eV and U = 2 eV, respectively. The red and brown balls represent the V and Te atoms, respectively. The cyan color indicates down-spin density, and the yellow color denotes up-spin density.

Our approach begins with a comparative analysis of the relative stability and magnetic configurations of bulk VTe_2_ (T versus T″) across various U values. As shown in Figure 1, our calculations reveal that bulk T″-phase VTe_2_ transits into the T phase upon full relaxation of the structures when the Hubbard U exceeds a critical value U_critical_ of ~2 eV. This transition is marked by a sharp change in atomic configuration, total magnetic moment, and volume. Conversely, the T″ phase is energetically favorable over T only at U = 0 eV (see Figure 1b). Considering the fact that VTe_2_ maintains its T″ phase at temperatures ≤ 480 K, we expect U = 0 eV to be a more reasonable value. In addition, the determined lattice parameters at U = 0 eV could well reproduce the crystal structure of bulk T″-phase VTe_2_. Specifically, the determined lattice parameters, *a* = 18.814 Å, *b* = 3.608 Å, *c* = 9.159 Å, and *β* = 134.5667° of the bulk T″-phase VTe_2_, are in favorable agreement with the experimentally determined parameters of *a* = 18.984 Å, *b* = 3.5947 Å, *c* = 9.069 Å, and *β* = 134.62° [49]. It is noted that the conventional cell is employed for direct comparison with the experimentally reported lattice parameters, whereas the primitive cell is utilized in the DFT simulations. Detailed information regarding the structure of the primitive cell of the bulk T″-phase VTe_2_, relaxed at U = 0 eV, is provided in Table 1.

The above results primarily delve into the impact of U on structural properties. In this section, we shift our focus to the influence of Hubbard U on magnetic properties. Due to the absence of the bulk T″ phase for U > 2 eV, all structures in this section are fixed to that of U = 0 eV. As shown in Figure 1c, the average magnetic moments (per VTe_2_ unit) of the bulk T″-phase VTe_2_ exhibits a striking change from nearly zero to >1 $\mu_{B}$ as the Hubbard U parameter increases from 0 to 4 eV. It noted that in Figure 1a, a significant change in magnetic moment observed at U > 2 eV is attributed to the phase transition from bulk T″ to the T phase. When U exceeds 2 eV, the T″ phase becomes highly unstable, transitioning into the T phase upon full structural relaxation. This transition leads to a significant increase in magnetic moment, as the T phase inherently possesses a larger magnetic moment compared to the T″ phase. Conversely, the magnetic moment variation in Figure 1c is solely influenced by the enhanced localization of electrons due to the increased Hubbard U value, as the structures remain fixed. In addition, our results reveal that the bulk T″-phase VTe_2_ manifests ferrimagnetic behavior only at U = 0 eV and it becomes ferromagnetic for all the other U values studied in this work (Figure 1d). As an example, the FM coupling at U = 2 eV is shown in Figure 1f. The respective magnetic moment for V1 and V2 are 0.53 $\mu_{B}$ and −0.16 $\mu_{B}$ for the ferrimagnetic coupling (Figure 1e). The results at U = 0 eV are quite close to the experimental observed antiferromagnetic (AFM) state [50]. The ferrimagnetic order obtained from our calculation is also qualitatively consistent with recent work by Won et al., which also confirmed the ferrimagnetic order to be the ground states [38]. Therefore, both the structural stability and the magnetic order of bulk VTe_2_ are accurately captured by GGA (U = 0 eV) rather than GGA + U.

**Figure 2:**
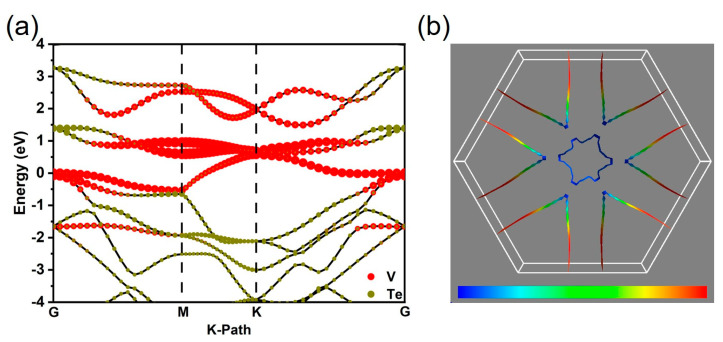
(**a**) Calculated band structure with projection on V and Te, and (**b**) Fermi surface of the T-phase VTe_2_ monolayer by using U = 0 eV. The Fermi is aligned to 0 eV. The color bar indicates the density of states at the Fermi level. The red (blue) color denotes high (low) density of states. The band structure could well match the energy band detected by the ARPES detection. Moreover, the experimentally detected Fermi surface could also be accurately reproduced [21,41,42].

In addition, our analysis extends to the VTe_2_ monolayer, in which we also ascertain that U = 0 eV remains a prudent selection. As shown in Figure 2, the utilization of U = 0 eV accurately reproduces the band structure and Fermi surface of the T-phase monolayer that was experimentally observed [21,41,42]. Notably, Wang et. al. have successfully reproduced the experimentally determined band structure (i.e., ARPES) of the T-phase monolayer with the GGA (U = 0 eV) method [41]. Consequently, based on the collective evidence, we confidently affirm that the GGA (U = 0 eV) method stands as a rational choice for modeling VTe_2_ properties, and all of the following results are obtained by calculations based on this choice.

### 3.2. The Structural, Electronic, and Magnetic Attributes of Monolayer T″-Phase VTe_2_

After choosing the optimum Hubbard U values, in the subsequent sections, our investigation will focus on the structural, electronic, and distinctly magnetic attributes of monolayer T″-phase VTe_2_, using the T-phase monolayer as a comparative benchmark. We prioritize the examination of ML T″-phase VTe_2_ for two primary reasons. Firstly, since bulk T″-phase VTe_2_ exhibits antiferromagnetism, it is reasonable to anticipate the persistence of magnetism in the monolayer counterpart. Secondly, the diminished geometrical symmetries in ML T″-VTe_2_, in contrast to the honeycomb V atom lattice in ML T-VTe_2_, are poised to introduce additional magnetic anisotropy; thus, promoting stable, long-range ferromagnetic order.

**Figure 3:**
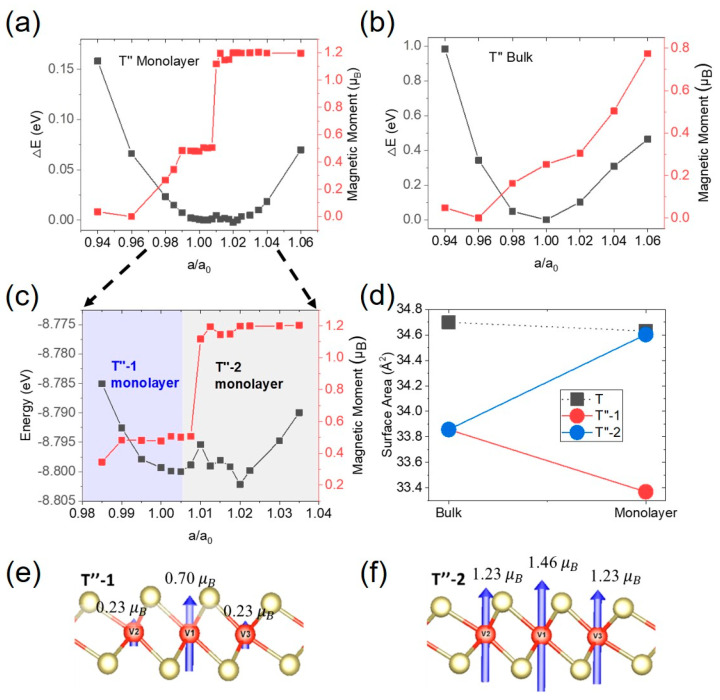
(**a**) Evolution of the relative energy and magnetic moment per formula unit in (**a**) ML T″-phase VTe_2_ and (**b**) bulk T″-phase VTe_2_ as a function of strain. (**c**) A closer examination of figure (**a**) by narrowing the ratio *a*/*a*_0_ into the range [0.98, 1.04]. It is noted that *a*_0_ = 18.687 Å and 18.814 Å for monolayer and bulk T″-phase VTe_2_. T″-1 and T″-2 denotes the two kinds of magnetic states. (**d**) The surface area of ML T″-1 and T″-2 VTe_2_ compared to bulk T″-phase VTe_2_. The surface area of ML and bulk T-phase VTe_2_ are given for comparison. The surface area is defined by the in-plane area of the unit cell with the *c* axis perpendicular to the VTe_2_ monolayers. (**e**) Magnetic order of ML T″-1 VTe_2_. Magnetic moments of V1, V2, and V3 are 0.70 $\mu_{B}$, 0.23 $\mu_{B}$, and 0.23 $\mu_{B}$, respectively. (**f**) Magnetic order of ML T″-2 VTe_2_. Magnetic moments of V1, V2, and V3 are 1.46 $\mu_{B}$, 1.23 $\mu_{B}$, 1.23 $\mu_{B}$, respectively. The red and brown balls represent the V and Te atoms, respectively. It is noted that the ground-state structures of ML T″ VTe_2_ have the lattice constant *a* of 18.687 (18.786) Å for T″-1 (T″-2).

First, we study the strain-dependent energetic and magnetic properties of both bulk and monolayer T″-phase VTe_2_. Within ML T″-phase VTe_2_, notably abrupt changes occur in both magnetic moment and energy as the in-plane lattice constant *a* traverses a critical threshold value around *a*_0_ (*a*_0_ = 18.687 Å) (see Figure 3a,c). In contrast, both the magnetic moment and energy of the bulk T″-phase VTe_2_ exhibit a smoother dependence on strain (see Figure 3b). Upon closer examination, we identify two distinct ferromagnetic states within ML T″-phase VTe_2_. Figure 3c denotes the two kinds of ferromagnetic states of ML T″-phase VTe_2_: the T″-1 state at the side of compressive and the T″-2 state at the side of tensile strain, respectively. Similarly, the magnetic moment of the middle V atoms (V1) is larger than the neighboring two V atoms (V2 and V3) for both the T″-1 and T″-2 states. However, there are big differences in the magnitude of the magnetic moments for these two states, that is, the magnetic moments of V1, V2, and V3 in ML T″-1 VTe_2_ are 0.70 $\mu_{B}$, 0.23 $\mu_{B}$, and 0.23 $\mu_{B}$, respectively (see Figure 3e), while the magnetic moment of V1, V2, and V3 in ML T″-2 VTe_2_ are 1.46 $\mu_{B}$, 1.23 $\mu_{B}$, and 1.23 $\mu_{B}$, respectively (see Figure 3f). As shown in Figure 3d, the fully relaxed structure of ML T″-1 VTe_2_ exhibits a smaller surface area than ML T″-2 VTe_2_, which is consistent with the result that the T″-1 (T″-2) state appeared in the compressive (tensile) area (see Figure 3c). The surface area of T″-2 is in close proximity to the ML T-phase VTe_2_, although the corresponding surface area of bulk T″-VTe_2_ is smaller than bulk T-phase VTe_2_.

**Figure 4:**
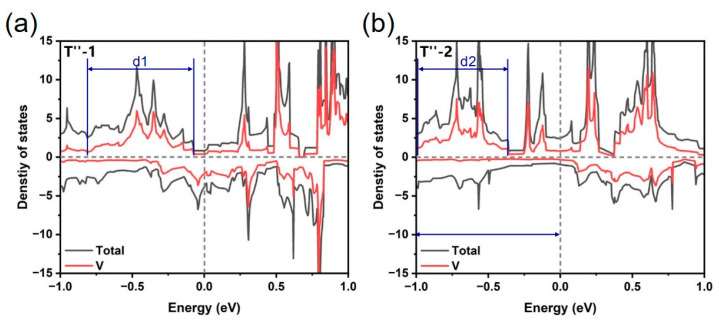
(**a**) A comparison of the spin-polarized total density of states (DOS) and projected density of states (PDOS) for (**a**) ML T″-1 VTe_2_ and (**b**) ML T″-2 VTe_2_. The Fermi level is aligned to 0 eV. d1 and d2 are the bandwidths of the *d* orbital around the Fermi level for ML T″-1 and T″-2 VTe_2_, respectively.

The above results suggest V atoms in T″-1 (T″-2) are more delocalized (localized), which is caused by the smaller (larger) V-V distance under compressive (tensile) strain. This could be reflected by the hopping energies (*t*) between the V atoms. A direct estimation of the hopping energies requires the tight binding method, which is out of the scope of this work, but the hopping energies are known to be proportionally linear to the bandwidth. Moreover, as shown in Figure 4, the bandwidths of the *d* orbital around the Fermi level for ML T″-1 (d1) and T″-2 VTe_2_ (d2) are 0.73 and 0.62 eV, respectively. Considering we are using the same U values for these two systems, the spin polarization should be inversely proportional to *t*, which is in line with the larger magnetism in T″-2. Despite the minor change in atomic configuration, the density of states for ML T″-1 and T″-2 VTe_2_ exhibit an evident difference. For example, the T″-1 has more spin-down density states located below the Fermi level than T″-2. Consequently, ML T″-2 VTe_2_ exhibits larger spins splitting between the spin-up and spin-down channels than that of the ML T″-1 VTe_2_ (see Figure 4). This observation concurs with the anticipation that the former would possess a significantly larger average magnetic moment than the latter. As T″-1 VTe_2_ is more energetically stable than T″-2 phase when no strain is applied, we will further study the magnetic properties of T″-1 VTe_2_ in the following section.

**Figure 5:**
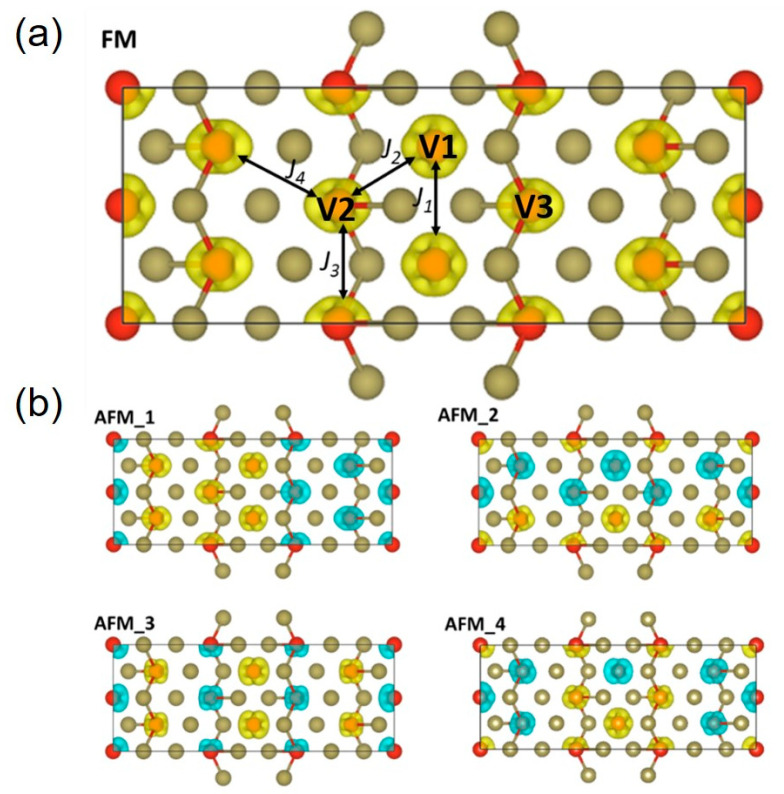
The (**a**) ferromagnetic and (**b**) four antiferromagnetic configurations adopted for T″-1 VTe_2_. The red and brown balls represent V and Te atoms, respectively. J_1_, J_2_, J_3_, and J_4_ are the four unique types of exchange energies between the nearest neighboring V-V exchange paths in ML T″-1 VTe_2_. The yellow and cyan colors denote the up-polarized spin density and down-polarized spin density, respectively. Note that the FM order is the ground state magnetic configuration.

To determine the magnetic ground state of the T″-1 VTe_2_, we examined one ferromagnetic and four antiferromagnetic configurations (refer to Figure 5). In Table 2 are the corresponding total energies, which have been decomposed into the nearest-neighboring V-V magnetic exchange energies (J_1_, J_2_, J_3_, and J_4_) and the energy from all the other contributions. Our findings validate that the ferromagnetic coupling represents the ground magnetic state of the T″-1 VTe_2_.

As shown in Table 2, the strongest magnetic interaction is the FM coupling (−20.93 meV per link) between the V2 atoms. Due to the symmetry, the same coupling is expected between the V3 atoms. Notably, the coupling between the V1 chains with either the V2 or V3 chains is also significant (−15.615 meV per link). On the contrary, the couplings between V1 atoms and between V2 and V3 chains exhibit an antiferromagnetic nature, albeit with orders of magnitude smaller than the FM couplings. Thus, we could expect that an overall FM coupling is preferred for the systems, a hypothesis that will be further corroborated by the explicit Monte Carlo simulations detailed below.

The strong coupling between magnetic moments does not necessarily lead to long-range magnetic ordering at a finite temperature in low-dimensional systems unless magnetic anisotropy is included in the system. In contrast to the structural isotropy along the lattices *a* and *b* for T-phase VTe_2_, the 3×1 reconstruction in T″-1 VTe_2_ breaks such symmetry, which may induce magnetic anisotropy in the system. Consequently, we performed simulations on the magnetic anisotropy energy (MAE) of both the ML T- and the T″-1 VTe_2_.

**Figure 6:**
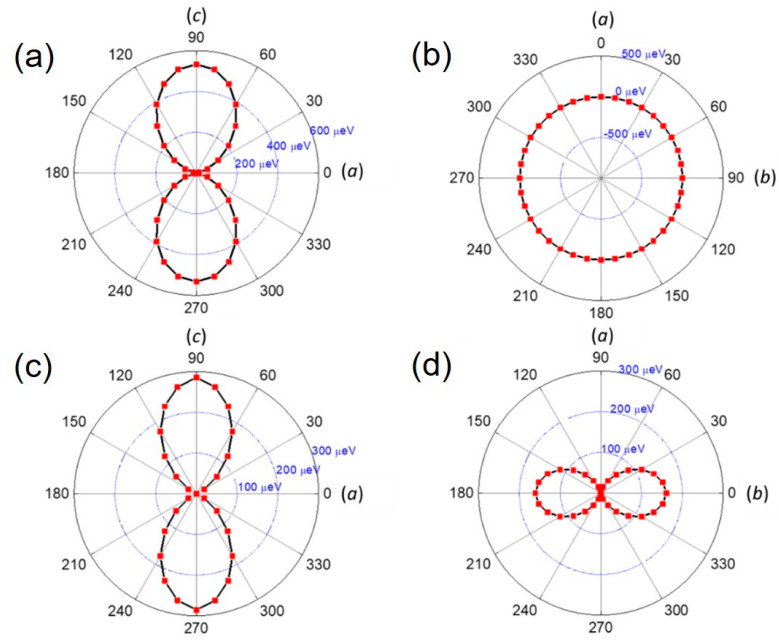
Magnetic anisotropy of ML T- and T″-1 VTe_2_. (**a**,**b**) illustrate the relative energy as a function of the spin rotated angles (red squares) for ML T-phase VTe_2_ in the *ac* plane (out of plane) and the *ab* plane (in plane), respectively. (**c**,**d**) illustrate the relative energy as a function of the spin rotated angles for ML T″-1 VTe_2_ in the *ac* plane and the *ab* plane, respectively. The black curves are a guide for the eyes.

As shown in Figure 6, the ML T-phase VTe_2_ exhibits an easy-plane anisotropy, with its magnetic moments preferring to stay in the *ab* plane (in-plane) rather than along the *c* direction, while the energy remains isotropic within the *ab* plane (see Figure 6a,b). On the contrary, apart from its inclination to remain in the in-plane over the out-of-plane direction, the ML T″-1 VTe_2_ also exhibits an anisotropy within the plane. Specifically, it demonstrates an easy-axis anisotropy perpendicular to the chain (along the *a* axis) and a substantial magnetic anisotropy energy of 165 μeV (Figure 6c,d). Therefore, ML T″-1 VTe_2_ promises to be a stable ferromagnetic material while ML T-phase VTe_2_ does not exhibit stable long-range ferromagnetic order at finite temperatures.

**Figure 7:**
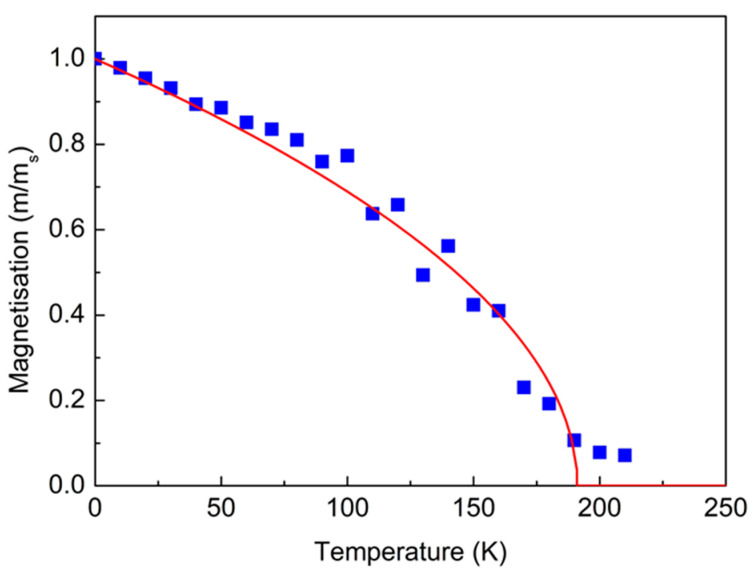
The temperature dependence of normalized magnetization for monolayer T″-1 VTe_2_ (blue solid squares). The red curve is the fitted Curie–Bloch equation in the classical limit from the simulated temperature-dependent magnetization.

To precisely determine the Curie temperature, we employed the Heisenberg spin Hamiltonian, which can consider the impacts of both spin–spin interactions and magnetic anisotropy on the temperature-dependent spin behavior of the monolayer T″-1 VTe_2_ system. This analysis was conducted through Monte Carlo Metropolis simulations utilizing the VAMPIRE software package [48]. Figure 7 illustrates the temperature-dependent magnetization obtained from these simulations. The simulated temperature-dependent magnetization was further fitted to the Curie–Bloch equation in the classical limit, $m\left(T\right)=(1-T/T_{c}{)}^{\beta }$, where $T_{c}$ is the Curie temperature and *β* is the critical exponent. As shown in Figure 7, there is large net magnetism at low temperatures, demonstrating the pinning of the magnetic moments to a specific direction due to the easy-axis magnetic anisotropy. However, the normalized magnetism progressively decreases with temperature, transitioning to a paramagnetic state at the critical Curie temperature $T_{c}$ of around 191 K. Notably, this Curie temperature significantly surpasses that of the CrI_3_ monolayer (45 K), confirming the stabilization of long-range collective magnetism in the T″-1 VTe_2_. This stabilization is attributed to the magnetic anisotropy stemming from periodic structural distortions within the material.

## 4. Conclusions

In this study, we conducted a systematic analysis of the correlation between structure and magnetism and their dependence on strain in monolayer (ML) T″-phase VTe_2_. By setting the Hubbard U parameter to 0 eV, we were able to replicate the experimentally observed properties of both bulk and monolayer T″-phase VTe_2_ accurately. Under applied strain, we identified two distinct ferromagnetic states in the monolayer T″-phase VTe_2_: the T″-1 state, which emerges under compressive strain, and the T″-2 state, which appears under tensile strain. These states are characterized by minor structural differences yet exhibit significant variations in their magnetic moments, with the T″-1 state displaying lower magnetic moments and the T″-2 state showing higher magnetic moments. Interestingly, in the absence of strain, the T″-1 VTe_2_ is energetically more favorable compared to the T″-2 phase.

Furthermore, our comparison of magnetic anisotropy between the T and T″-1 phases of VTe_2_ reveals that periodic lattice distortion in the T″-1 phase leads to in-plane anisotropy, making the T″-1 VTe_2_ an easy-axis magnetic material. Monte Carlo simulations support our findings, highlighting a remarkably high Curie temperature of approximately 191 K for T″-1 VTe_2_. These insights enhance our understanding of the VTe_2_ system. In addition, our study presents a new and promising direction for stabilizing low-dimensional magnetism, which holds promise for spintronics applications due to its enhanced stability of ferromagnetic ordering in thin layers, facilitating efficient manipulation and control of spin-polarized currents for advanced electronic devices.

## Figures and Tables

**Table 1:** The structure parameters of bulk T″-phase VTe_2_ relaxed at U = 0 eV. The first three rows present the lattice vectors *a*, *b*, and *c*, with the three columns indicating the Cartesian components of the lattice vectors. The subsequent nine rows detail the ion positions, with the three columns specifying the fractional coordinates relative to the three lattice vectors.

**Lattice**	***x***	***y***	***z***
*a*	7.4403	0	0
*b*	6.5837	3.4660	0
*c*	−10.8930	−2.6921	8.5749
**Atom**	***a***	***b***	***c***
V1	0.3468	0.3468	0.70819
V2	0.6532	0.6532	0.29181
V3	0	0	0
Te1	0.62794	0.62794	0.98386
Te2	0.37206	0.37206	0.01614
Te3	0.08852	0.08853	0.38103
Te4	0.91147	0.91148	0.61897
Te5	0.70404	0.70404	0.69656
Te6	0.29596	0.29596	0.30344

**Table 2:** The summary of the different magnetic configurations and the deduced magnetic exchange energies. FM, AMF_1, AMF_2, AMF_3, and AMF_4 are the magnetic configurations and J_1_, J_2_, J_3_, and J_4_ are the exchange energies between the nearest neighboring V-V, as shown in Figure 5. E_tot_ is the total energy of T″-1 VTe_2_ in different magnetic configurations. The integrals in the first five rows are the number of the respective exchange couplings in specific the magnetic configurations. Positive values indicate the ferromagnetic coupling while negative values are for AFM coupling. The values in the last row are the deduced exchange energies from the different magnetic configurations.

	J_1_ (meV/Link)	J_2_ (meV/Link)	J_3_ (meV/Link)	J_4_ (meV/Link)	E_tot_ (eV)
FM	8	16	4	8	−194.33
AFM_1	−8	0	−4	0	−193.99
AFM_2	8	0	4	8	−194.08
AFM_3	8	−16	4	−8	−193.90
AFM_4	8	0	−4	−8	−193.99
	2.77	−15.61	−20.93	4.85	

## Data Availability

Data are contained within the article. The data that support the findings of this study are available on request from the corresponding author.
